# Statistical optimization of a sustainable fertilizer composition based on black soldier fly larvae as source of nitrogen

**DOI:** 10.1038/s41598-022-24964-2

**Published:** 2022-11-28

**Authors:** Silvia Barbi, Monia Montorsi, Lara Maistrello, Matteo Caldironi, Luisa Barbieri

**Affiliations:** 1 Department of Sciences and Methods for Engineering, University of Modena and Reggio Emilia, Via Amendola 2, 42122 Reggio Emilia, Italy; 2 Interdepartmental Center for Applied Research and Services in Advanced Mechanics and Motoring, INTER-MECH-Mo.Re., University of Modena and Reggio Emilia, Via P. Vivarelli 10/1, 41125 Modena, Italy; 3 Department of Life Sciences, University of Modena and Reggio Emilia, Via Amendola 2, 42122 Reggio Emilia, Italy; 4 Interdepartmental Center for Agri-Food Biological Resources Improvement and Valorization, BIOGEST-SITEIA, University of Modena and Reggio Emilia, Piazzale Europa 1, 42124 Reggio Emilia, Italy; 5 Department of Law, University of Modena and Reggio Emilia, Via San Geminiano 3, 41121 Modena, Italy; 6 Department of Engineering “Enzo Ferrari”, University of Modena and Reggio Emilia, Via Vivarelli 10/1, 41125 Modena, Italy

**Keywords:** Biomaterials - proteins, Environmental impact, Agroecology

## Abstract

In the present work, a statistical optimization of a sustainable coating for core–shell NPK (Nitrogen–Phosphorus–Potassium) fertilizers was investigated. The environmental green coating was enriched in nitrogen using a biomass and renewable source, namely the nitrogen rich fraction of black soldier fly larvae (BSFL) (*Hermetia Illucens*, Diptera: Stratiomyidae) reared on vegetable waste. A rational approach was proposed with the aim of calculating the best formulation of the coating, considering both its manufacturing behavior, such as adhesion to the core, and its physical properties, such as homogeneity or plasticity. From a circular economy perspective, together with the nitrogen-rich fraction from BSFL (from 51 to 90 wt.%), water and glycerol were considered for the coating formulation in different proportion: from 10 to 32 wt.% and from 0 to 17 wt.% respectively. The Design of Experiments technique was implemented to limit the total number of tests for the coating formulation (18 tests). ANOVA was employed, with the aim of obtaining mathematical models to derive a better precise and objective formulation. The results show that the use of glycerol can be avoided, as well as only a limited amount of water (11 wt.%) is necessary to obtain an optimized coating formulation, thereafter, satisfying the more relevant technological and physical properties for the coating manufacturing.

## Introduction

The global population is expected to reach 9.7 billion in 2050 and 10.9 billion in 2100^1^. This growth will have to deal with climate change and its effects on global food production, as by the end of the twenty-first century, climate change is predicted to transform between 1.8% and 4.6% of global land into arid lands, affecting over 270 million people^2^. Currently, cultivated land will also suffer increasing pressure caused by urbanization^3^ and, consequently, by more intense exploitation^4^. Furthermore, activities related to agriculture and land use accounted for 71% of greenhouse gas (GHG) emissions from the food production system in 2015, therefore it is important to strengthen the sustainability and efficiency of the agricultural system^5,6^.

In this context, fertilizers, and in particular “environmentally friendly fertilizers “ (EFFs), are one of the main pillars of modern agriculture, as they allow an enormous increase in crop production per unit of land, providing plants with the main nutrients necessary for their growth^7–10^. To produce EFFs, natural, naturally derived, or organic materials are generally preferred, as they have a lower impact on the environment, are readily available, and can help avoid or limit pollutants in the soil, compared to petroleum-derived polymers^11–13^. In addition, they may have other positive characteristics, such as increasing the soil organic matter content or enriching the soil with a particular nutrient^11,14^. However, the materials used to produce EFFs show several shortcomings that hinder their spread, e.g., production processes are often complicated or expensive, while environmental conditions have unknown effects on actual nutrient release patterns^12,15,16^.

Among the macronutrients of plants, nitrogen is one of the most demanding, as urea-based fertilizers are characterized by a loss of between 40 and 70%^17^. This loss is the cause of several pollution processes, as groundwater is contaminated with leached nitrates and the atmosphere is exposed to both NH_3_ volatilization and GHG emissions, such as N_2_O^18–21^. On the other hand, ammonia-based fertilizers are extremely harmful to the environment as 2000 kg of CO_2_ are generated for every 1000 kg of obtained NH_3_, and the entire production process depends on the use of natural gas^22^. In this context, an alternative source of nitrogen is needed, and a possible source can be identified in the conversion of organic waste materials, while at the same time partially solving the ecological problems deriving from the disposal of organic waste^23–28^. Among the biological or chemical methods to convert organic waste, the use of black soldier fly larvae (BSFL) (*Hermetia Illucens*, Diptera: Stratiomyidae) is considered an efficient and safe bioconversion tool for their treatment^29–31^. In fact, when the valorization of by products or waste is considered, it is quite often necessary to exploit their value in terms of macronutrients through conversion, due to physical (shape and dimension) and biological–chemical (availability of the macronutrients) limits. BSFL have been suggested for efficient biowaste recycling as their action leads to a marked reduction in initial waste weight (up to 68% of the initial dry mass^32^), inhibition of pathogens, such as Salmonella^33,34^, reduction of GHGs emissions^35^ and odorous emissions^36^ when compared with standard composting procedures. In addition, BSFL have a highly efficient feed conversion rate, leading to a valuable biomass rich in nitrogen (30–50 wt.%) and lipids (21–40 wt.%)^37^, whose composition varies according to the employed organic waste. or fermentation strategy trough specific additives, such as industrial flocculants, and device, such as artificial light^38–42^.From this, it emerges that the nitrogen-rich BSFL fraction, containing mainly protein and chitin, could represent as a valuable source of organic nitrogen useful for crop growth, and thereafter for the production of cheap and sustainable organic fertilizers^43^. However, current European legislation places some limits on the type of organic substrates to be used for bioconversion by BSFL, preventing the use of manure and any substrate formally recognized as “waste” as feed for animals^44^. In particular, fruit and vegetable residues seem to qualify as by-products pursuant to Article 184-*bis* of the Italian legislative decree 152 of 2006^45^. Indeed, such residues seem to possess the characteristics necessary to comply with the four conditions required by law to qualify a residue as a by-product. The four conditions are: (a) the residues originate from a production process of which they are an integral part and whose primary purpose is not the production of such residues; (b) further use of the substance or object is certain ; (c) the substance or object can be used directly without any further processing other than normal industrial practice; (d) further use is lawful, i.e. the substance or object fulfills all relevant product, environmental and health protection requirements for the specific use and will not lead to overall adverse environmental or human health impacts. If the residue meets the four conditions, therefore it can be qualified as by-product and can be freely reused without the need for permits and without being subject to the waste control and traceability regime. Moreover, the fruit and vegetable residues employed in this work seem to be able to fall under the definition of ‘feed’ (or ‘feeding stuff’) in Regulation (EC) No. 178/2002 (Art. 3(4)): «any substance or product, including additives, whether processed, partially processed or unprocessed, intended to be used for oral feeding to animals». For this reason, these residues cannot be made up of «solid urban waste, such as household waste» due to the express prohibition in Regulation (EC) No. 767/2009, Art. 6, Annex III (no. 6), however they can only derive from industrial activities (EUROPEAN PARLIAMENT, 2009; EUROPEAN PARLIAMENT 2002).

This work addresses the problem of alternative nitrogen sources in the context of EFFs, as there is a growing need to find sustainable sources and processes to obtain macro-nutrients for agricultural uses in a circular economy perspective. In this terms fiber food has been avoided in order to avoid any specific addition of chemical^47^ However, these sources must be as cheap as possible to compete with conventional fertilizers and comply with countries’ legislative limits. Therefore, in this work, different nitrogen-rich fractions obtained from the conversion of organic waste by BSFL were investigated, in order to optimize their manufacturing conditions for the production of a fertilizer coating. A bioconversion treatment of waste was needed in order to comply physical (dimension decreasing and homogeneity increasing) but also biological (increment of the available Nitrogen content and decreasing of possibly pathogens amount) requirements for coating manufacturing, In particular, a tailored formulation of a coating suitable for core–shell EFFs was derived through statistical methods to be applied over an inorganic core enriched in phosphorus and potassium, and previously optimized^48^. As an innovation from consolidated literature and unlike previous work, to comply with European legislation, the BSFL were reared on vegetable by-products from agri-food industries^49^. A rational approach was used to plan the experiments, using Design of Experiments (DoE) techniques, and a multivariate analysis of the variance (ANOVA) was applied to the data, to avoid the intrinsic limitations of the One-factor-At-Time (OFAT) approach, with the aim of obtaining mathematical models of correlation between the formulation of the coating and its performance. This numerical approach was applied to optimize the formulation and production of the coating in an industrial scale-up perspective, to improve the production process of an NPK (Nitrogen–Phosphorus–Potassium) fertilizer while also improving its economic and environmentally friendly production.

## Materials and methods

### Black soldier fly larvae rearing

The BSFL were provided by the Laboratory of Applied Entomology—BIOGEST-SITEIA, Department of Life Science, University of Modena Reggio Emilia, where a permanent BSF colony is reared as described by Macavei et al.^50^, in compliance with the appropriate laws and institutional guidelines. The BSFL were grown on a mix of vegetable substrates (Table 1) representative of processing by-products from different local agro-industrial chains which had also been used for a previous study aimed at optimizing the carotenoid content in the larvae (Leni et al.)^51^. The vegetable by-products were mixed in constant proportions to maintain the most homogeneous formulation and placed in glass boxes (40 × 30 × 20 cm LxWxH) inside a climatic chamber with constant temperature (27 ± 1 °C) and relative humidity (65 ± 5%). BSFL were initially placed as second/third instar (average weight 0.004 ± 0.001 g/larva) and collected before the prepupal stage, when they reached a weight of about 0.16 g/larva (approximately 8 days). Finally, the BSFL were frozen and stored at -20 °C until their use.

**Table 1:** Vegetable substrate composition for BSFL growth (wt.%).

Vegetable by-products	wt.%
Carrots	17.4
Apples	4.9
Kiwi	2.7
Tomatoes	1.6
Fava beans and pods	1.2
Peanuts and seeds	1.2
Chickpeas	0.5
Beans	18.5
Bran	4.6
Water	47.4

### Obtainment of the nitrogen-enriched fractions from BSFL

**Figure 1:**
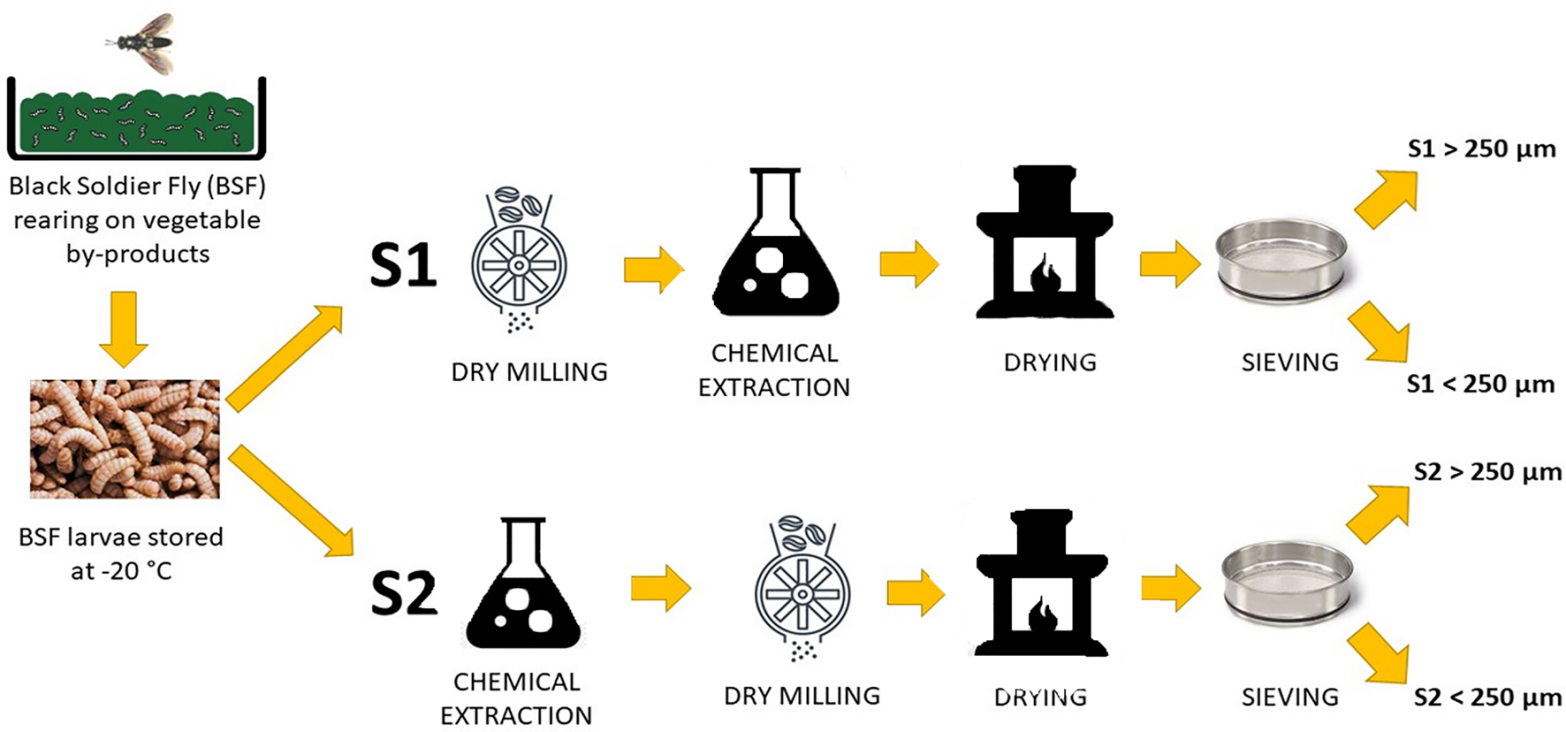
Methods for obtaining nitrogen-rich fractions.

The first nitrogen-enriched fraction (or N-enriched fraction), called S2, was extracted from frozen BSFL following the procedure reported in a previous study^52^. Briefly, petroleum ether (ACS reagent, boiling point 40–60 °C, reagent grade, CAS 101316–46-5) was employed to extract the lipid fraction of the BSFL biomass by immersion and mechanical mixing. The residual part of the BSFL biomass, containing the nitrogen-rich fraction derived both from chitin and proteins, was dried for 24 h at 60 °C (SassuoloLab—Italy) and then ground with an analytical dry mill for 5 min at 20,000 rpm (A10—IKA-Werke GmbH & Co. Germany) to obtain a homogeneous powder. Finally, the S2 fraction was sieved (SassuoloLab—Italy) to obtain two different particle sizes of the powder (one below 250 μm and one above 250 μm) with the aim of comparing the influence of the different particle sizes on the formation of the coating. According to the purpose of the present study, a further procedure was used to obtain the nitrogen-rich fraction from BSFL with the aim of reducing the obtaining times (mainly due to the drying phase) and, possibly, increasing the nitrogen content. This new procedure consists of grinding the larvae before mixing with petroleum ether to increase the reaction surface of the insect purée (Fig. 1). This nitrogen rich fraction was called S1. As expected, this procedure was capable to decrease the drying time from 24 h, needed for S2, to 8 h. After the drying phase, S1 was then sieved to obtain two fractions with different particle sizes, with the same procedure employed for S2.

### Design of experiment approach to coating formulation

A Design of Experiments (DoE) approach, i.e. a statistical and rational technique for deriving mathematical models, was employed to set the minimum number of experiments necessary to save time and raw materials, as well as to avoid information loss^53^. A combined design was implemented with the aim of deriving correlations, not only regarding the quantities of compounds in the formulation of the coating (Mixture Design) but also their typology (Factorial Design). Three factors were considered for the mixture design: fraction from BSFL “BSF” (from 51 to 90 wt.%), water (from 10 to 32 wt.%) and glycerol (from 0 to 17 wt.%). The ranges of each compound were chosen by considering another application in agriculture of fractions from BSFL reported in literature^54^. For the factorial part of the combined design, two categorical factors were considered, with two levels each, relating to the type of nitrogen-rich fraction: particle size (< 250 or > 250) and method of obtaining (S1 or S2). The other variables that occurred in the process and were not specifically considered in this study, such as temperature and humidity, were kept constant during all the tests, according to the procedure described in paragraphs 2.1, 2.2 and 2.4. The Design Expert 13.0 (Stat-Ease, Minneapolis, MN, U.S.A.) code was used both to set up the experimental plan and to analyze the results. Due to the large number of factors, a combined fractional factorial design was selected, as fractional designs are a specific statistical tool aiming to select a limited number of experiments that are indispensable to derive reliable mathematical models^53^. A total of 18 experiments were collected in the combined factorial design and performed, including repetitions for pure error estimation (Table 2). The central points, considered as the arithmetic mean of the factors’ levels, were included to investigate the presence of curvature in the data analysis. All the experiments (runs) were carried out randomly to avoid the presence of systematic errors, following the experimental method reported in paragraph 2.4.

**Table 2:** Experimental plan obtained with Design Expert 13.

Run	BSF (wt.%)	Glycerol (wt.%)	Water (wt.%)	N-fraction obtainment	Particle size
1	51	17	32	S1	< 250 μm
2	61	13.5	25.5	S1	< 250 μm
3	71	10.1	18.9	S1	< 250 μm
4	71	0	29	S1	< 250 μm
5	51	17	32	S2	< 250 μm
6	61	13.5	25.5	S2	< 250 μm
7	71	10.1	18.9	S2	< 250 μm
8	71	0	29	S2	< 250 μm
9	51	17	32	S1	> 250 μm
10	61	13.5	25.5	S1	> 250 μm
11	71	10.1	18.9	S1	> 250 μm
12	71	0	29	S1	> 250 μm
13	51	32	17	S2	> 250 μm
14	80	20	0	S2	> 250 μm
15	71	18.9	10.1	S2	> 250 μm
16	71	29	0	S2	> 250 μm
17	90	10	0	S2	> 250 μm
18	61	25.5	13.5	S2	> 250 μm

The objective of the study was to evaluate the workability of the coating employing different nitrogen-rich fractions in the formulation of the coating. Subsequently, three responses of the coating were evaluated: (1) the homogeneity of the applied layer on the core surface (Homogeneity); (2) the difficulty of covering the core (Adhesion); (3) the plastic behavior of the coating paste (Plasticity). The evaluation of these three responses was carried out through a consensual panel test, and the scores obtained were recorded as responses and then analyzed using statistical methods^53^. Each category was assigned a score from 0 to 3 or 0 to 5 as shown in Table 3. The panel test was carried out using the blind judgments of five people.

**Table 3:** Panel test evaluation and target for the response variables.

Score	Homogeneity	Adhesion	Plasticity
0	Coating impossible to obtain	Adhesion completely failed	Paste similar to a wet powder
1	Coating with macro defects	Adhesion needs a strong manual fixing	Paste almost ideal
2	Coating with micro defects	Adhesion needs a medium manual fixing	Ideal paste for manual application
3	Coating without defects	Adhesion needs a limited manual fixing	Sticky paste
4	–	–	Paste almost liquid
5	–	–	Paste totally liquid
Target =	To maximize	To maximize	Score = 2

Analysis of Variance (ANOVA) was used to highlight the cause-effect relationship between the coating formulation and the response related to the workability of the coating on the fertilizer’s core. The main assumptions of ANOVA are that each input factor is independent of each other, normally distributed, and that the variation of the response can be decomposed into different components to evaluate the effect of each factor, their interactions, and the experimental error (or unexplained residual)^53^. The p-value, related to the F-test, is the statistical parameter used to evaluate the significance of the model and of each factor, and represents the probability that the considered model or factor is significant (p-value < 0.05) or not under the same experimental conditions^55^. Lack of fit test was also considered, as a significant lack of fit means that the variation of the design points about their predicted values is much larger than the variation of the replicates about their mean values, thereafter, a not significant lack of fit is desired. The quality of fit in terms of regression analysis and the predictive power of the model were assessed using the R^2^, Adjusted R^2^ and Pred-R^2^, respectively. R^2^ is the proportion of the variance in the dependent variables that is predictable from the independent variables, Adjusted R^2^ is a corrected R^2^ in proportion to the number of tests employed (thereafter attempting to correct any overestimation of the R^2^ due to the increasing number of effects included in the model) , and Pred-R^2^ is analogous to R^2^ but associated with predicted values^56^.

Finally, a global desirability function was calculated to provide the most desirable mixture and factorial factors, taking into account all the responses analyzed simultaneously^57^. Each response is weighed according to its specific target (Table 3) depending on how much each response must match the tailored purpose, and then combined using a mean. The desirability function range is from 0 to 1, where the lowest value (0) represents a completely undesirable combination of independent factors, and, conversely, the highest value (1) indicates a completely desirable or ideal combination of them.

### Coating manufacturing

The core of the investigated EFFs fertilizer is a porous ceramic granule, spherical with a diameter around 1.5 cm, as reported in a previous work^48^. In short, the core is composed of clay and local industrial wastes such as pumice scraps and spent coffee grounds, enriched in potassium (K) and phosphorus (P) in the form of K_2_CO_3_ and cattle bone flour ash, respectively. To add the nitrogen-rich coating on the surface of the core, it is necessary to mix a homogenous paste with other compounds and apply it manually to the core. For this reason, glycerol (GL, Sigma-Aldrich, reagent grade: 99%) was tested as a plasticizing agent, while water was employed as a solvent. For each experiment reported in Table 2 three granules were made to evaluate the reproducibility of the result and the values reported in Table 4 are the averages among these three repetitions.

**Table 4:** Elemental analysis of the nitrogen-rich fractions.

	N %	C %	H %
S2	8.56	45.17	7.04
S1	7.06	46.35	6.92

### Characterization

The nitrogen-rich powder fractions were characterized through chemical elemental analysis, FT-IR and morphological analysis to evaluate their chemical, physical and structural properties. In particular, the elemental analysis was performed through an elemental analyzer (Thermo Fisher, FLASH 2000). The analysis of the morphology and particle size of the powders was performed by using a scanning electron microscope (ESEM, FEI XL-30). Structural interactions within the nitrogen-rich fractions were assessed by attenuated total reflectance Fourier transform infrared (FTIR-ATR) spectroscopy. The FTIR-ATR spectra were obtained with a Bruker Vertex 70 spectrophotometer in the range of 400–4000 cm^−1^, with 4 cm^−1^ resolution and 30 scans. The best and worst coatings obtained after the statistical analysis were characterized in terms of structure and morphology. For the latter, an optical microscope at a magnification of 8X (Leica DM3XL) and a scanning electron microscope (ESEM, FEI XL-30) were employed. Structural analysis was performed through FT-IR spectroscopy with the same procedure used for the nitrogen-rich powder.

## Results and discussion

### Characterization of the nitrogen-rich fractions

The mass efficiency conversion of both nitrogen-rich fractions, S1 and S2, reports a recovering of approximately 40% of the total BSFL mass as a nitrogen-rich fraction, and this result is in line with previous literature. In fact, as reported in a previous study, BSFL contains: 32 wt.% of proteins, 37 wt.% of lipids, 19 wt.% of minerals, 9 wt.% of chitin and 3 wt.% of humidity^52^. Table 4 shows the elemental analysis of the two nitrogen-rich fractions, suggesting that the method employed for S2 is the most favorable to recover the highest nitrogen amount. It is worth noting that both the BSFL fractions shows a nitrogen content well above other sources of nutrients, reported in literature as possible fertilizers, such as duckweed plant or earthworm reared on organic substrate^13,58^ (Fernandez Pulido et al., 2021; Lv et al., 2021b).

**Figure 2:**
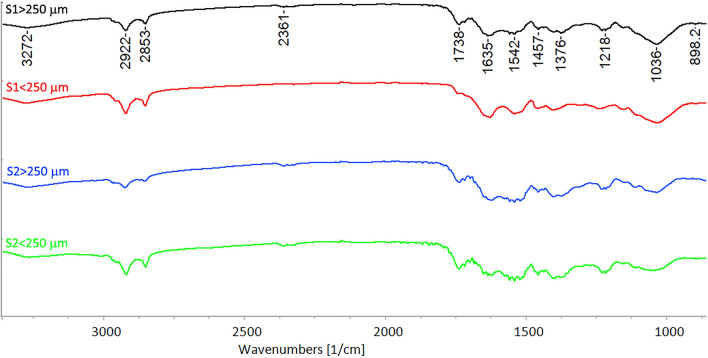
Normalized FT-IR transmittance spectra of the nitrogen-rich fractions.

Figure 2 shows the normalized transmittance spectra of the four nitrogen-rich fractions obtained after sieving, since FTIR spectroscopy is commonly used to identify the crystalline form (α, β or γ) of chitin, as well as presence of proteins and lipids^59^. The characteristic wavelengths of chitin, that have been repeatedly reported and are independent of the biological source, are: 3273 cm^−1^ (N–H stretch); 1630 cm^−1^ (C=O stretch); 1540 cm^−1^ (N⎼H bend, C⎼N stretch); 1450 cm^−1^ (CH_2_ bending and CH_3_ deformation); 1380 cm^−1^ (CH bending, CH_3_ symmetrical deformation); 1030 cm^−1^ (C⎼O⎼C asymmetric stretch in phase ring) and 890 cm^−1^ (CH ring stretch). According to literature, the carbonyl group of α-chitin is involved in two hydrogen bonds, one intramolecular (between the carbonyl group and ⎼CH_2_OH) that has a peak at around 1630 cm^−1^, and one intermolecular (between ⎼NH⎼ and the carbonyl group) at 1660 cm^−1^. β-Chitin only exhibits one signal at 1650 cm^−1^ as a result of the weaker intramolecular hydrogen bond, and γ chitin also shows a less pronounced band at 1660 cm^−1^ and a clear and sharp at 1620 cm^−1^.^59–62^ In the current case (Fig. 2) a close similarity between the transmittance spectra of all the four nitrogen-rich fractions and in particular the prominence of α-chitin can be noted. Indeed, as shown in Fig. 2, all the spectra showed bands around 1635 cm^−1^. Similar results have been reported in the literature on the crystalline origin of isolated chitin in different stages of black soldier fly (larvae, prepupa, puparium, and adults)^63,64^. The presence of a band at around 1540 cm^−1^ confirms the protein content in this fraction, since this band is due to the stretching vibrations of the peptide bond (C=O)^65^. Only less pronounced bands at 2922 cm^−1^ and 2853 cm^−1^, linked to CH_2_ asymmetric stretching and CH_2_ symmetric stretching, respectively, showed traces of lipids^66^.

**Figure 3:**
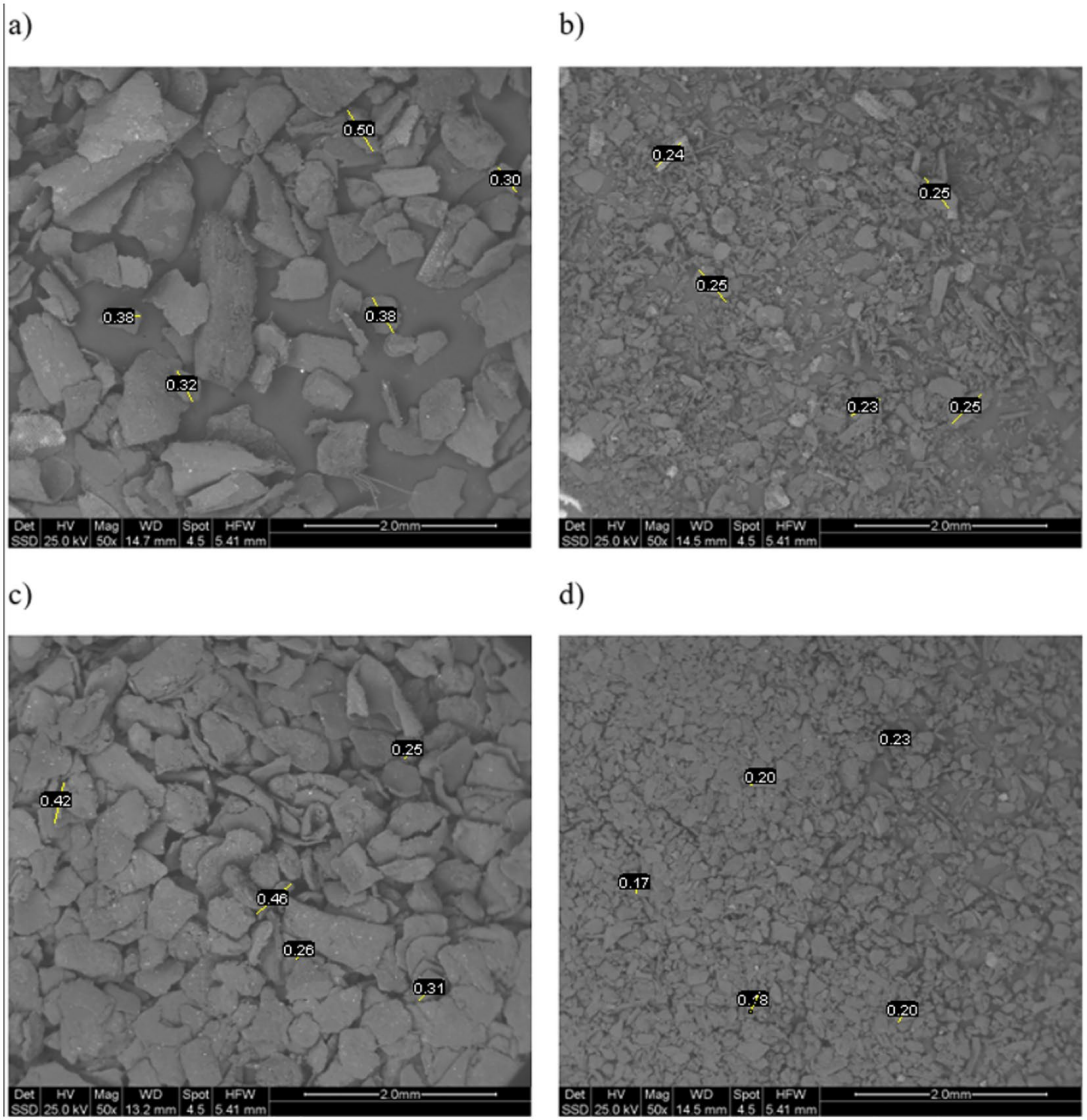
ESEM micrograph of the backscattered electrons at 50X all the measure are expressed in mm: (**a**) S1 > 250, (**b**) S1 < 250, (**c**) S2 > 250, (**d**) S2 < 250.

**Figure 4:**
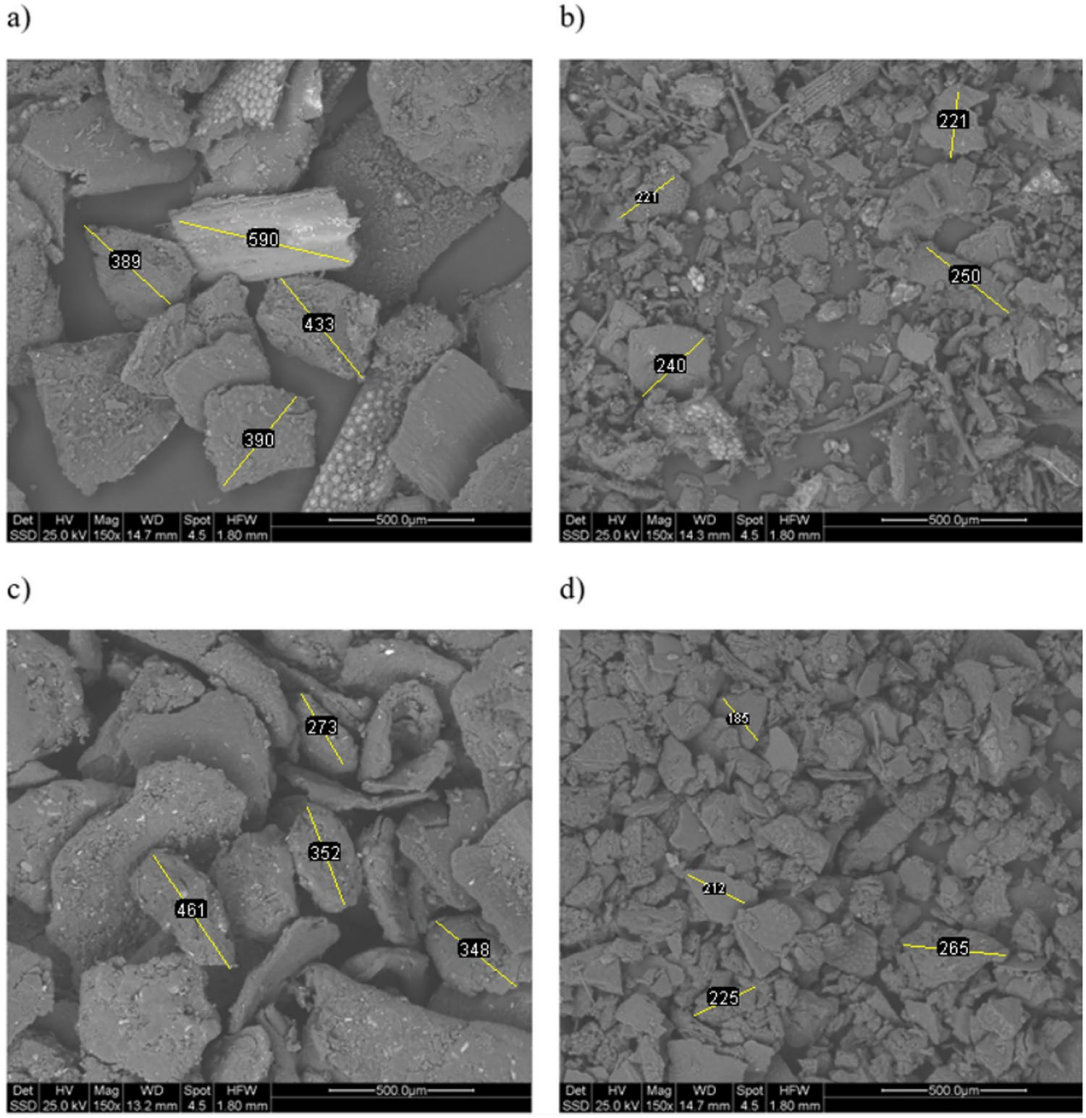
ESEM micrograph of the backscattered electrons at 150X, all the measure are expressed in μm: (**a**) S1 > 250, (**b**) S1 < 250, (**c**) S2 > 250, (**d**) S2 < 250.

**Figure 5:**
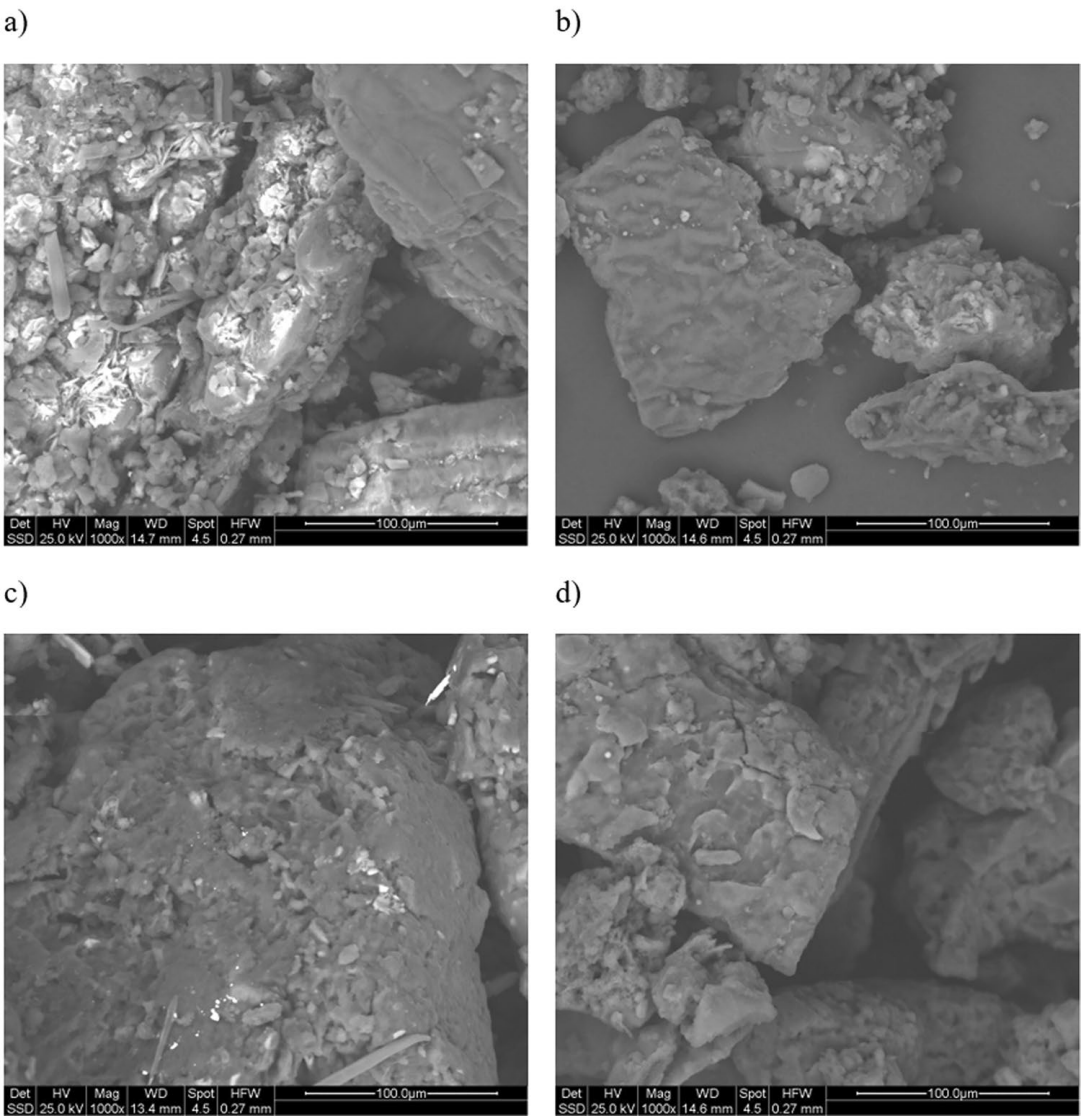
ESEM micrograph of the backscattered electrons at 1000X: (**a**) S1 > 250, (**b**) S1 < 250, (**c**) S2 > 250, (**d**) S2 < 250.

Regarding the physical properties, in Figs. 3, 4 and 5, ESEM micrographs at different magnifications were shown in order to study the physical morphology of the powders. From these figures the effectiveness of the sieving procedure can be assessed as a strong difference between fractions below and above 250 μm can clearly be seen, depending on the sieved used (Fig. 4). In addition, from Fig. 3a and Fig. 3c a strong difference is observed in terms of powder morphology as a function of the different obtaining process. In fact, a more regular and round particle shape characterizes the S2 fraction. This difference can also be slightly seen in the fractions below 250 μm (Fig. 3b,d). Finally, at higher magnification (Fig. 5) a similar surface porosity can be observed taking in account all four BSFL fractions investigated. From these preliminary considerations, relating only to the powders of the nitrogen-rich fractions, it seems that the S2 fraction could be the more favorable to be used for the coating manufacturing. First, a greater amount of nitrogen than S1 was evaluated through elemental analysis (Table 4). Furthermore, it should be noted that this amount of nitrogen (8.56%) is even greater than the one reported in literature and related to BSFL bioconversion of poultry manure (7.90%)^49^. Secondly, as shown by the ESEM characterization, S2 has the more regular particle morphology, probably leading to a more homogeneous coating than the one which could be obtained from S1.

### Statistical analysis

The 18 experiments detailed in Table 2 were carried out and rated through the panel test to evaluate their quality, the results are reported in Table 5, as well as images taken under the optical microscope for each experiment. From a preliminary observation of the coating through the optical micrographs, it is clear that strong differences occur in terms of coating production depending on the mixture and the factorial variables. Thereafter, a statistical evaluation is required to assess the influence of each parameter on the selected responses.

**Table 5:** Complete experimental plan.

RUN	Adhesion	Homogeneity	Plasticity	Optical micrographs 8X 1 cm
1	3	3	2	
2	3	3	2	
3	0	0	1	
4	1	2	1	
5	1	1	3	
6	1	1	3	
7	2	3	3	
8	3	3	2	
9	3	2	1	
10	2	1	1	
11	2	0	1	
12	1	1	1	
13	0	0	5	
14	2	2	3	
15	2	2	4	
16	2	2	4	
17	3	3	2	
18	1	1	4	

The ANOVA results were presented in Table 6 in which the quantification of the significance (p-value) of each model and that of the related quality parameters (R^2^, Adjusted- R^2^ and Pred-R^2^) were reported. In addition, in Table 7 the estimate of the coefficients was reported, in order to clearly evaluate which mixture or factorial factors are more relevant from a statistical point of view.

**Table 6:** ANOVA results.

Response	p-value	Lack of fit	R^2^	Adjusted R^2^	Pred-R^2^
Adhesion	0.0014	0.342	0.77	0.68	0.43
Homogeneity	0.0034	0.763	0.68	0.58	0.47
Plasticity	< 0.0001	0.574	0.93	0.90	0.81

**Table 7:** Coefficient estimation (coded factors). Standard error = 5%.

	Adhesion	Homogeneity	Plasticity
BSF	1.20	0.18	-0.61
Glycerol	2.06	-4.32	12.83
Water	3.23	7.57	-13.15
BSF—N-fraction obtainment	2.93	3.82	1.26
Glycerol—N-fraction obtainment	−7.28	–	–
Water—N-fraction obtainment	−4.48	−8.49	–
BSF-water	–	–	27.97

**Figure 6:**
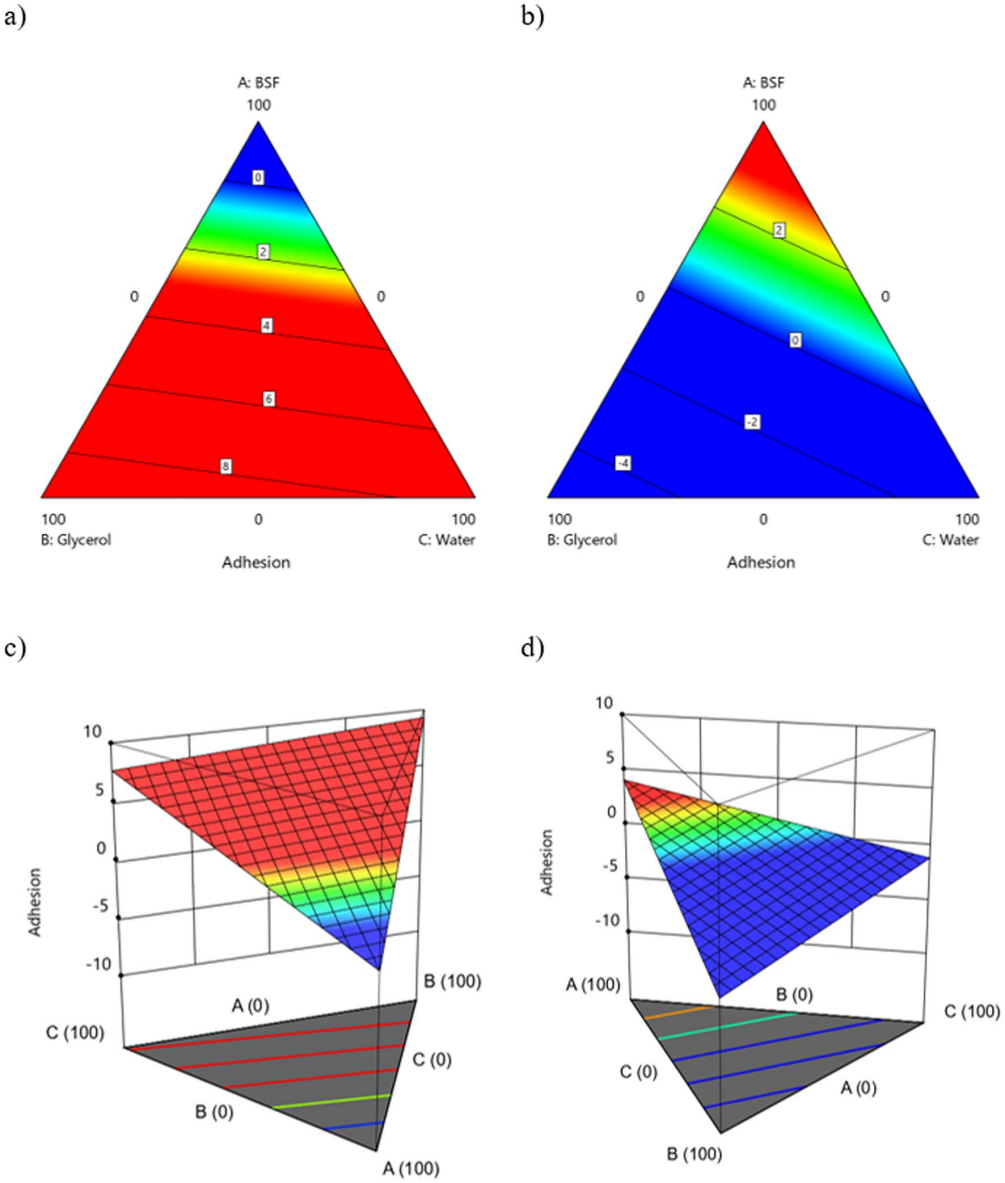
Contour plots for the response Adhesion (**a**) S1-2D; (**b**) S2-2D; (**c**) S1-3D; (**d**) S2–3D.

**Figure 7:**
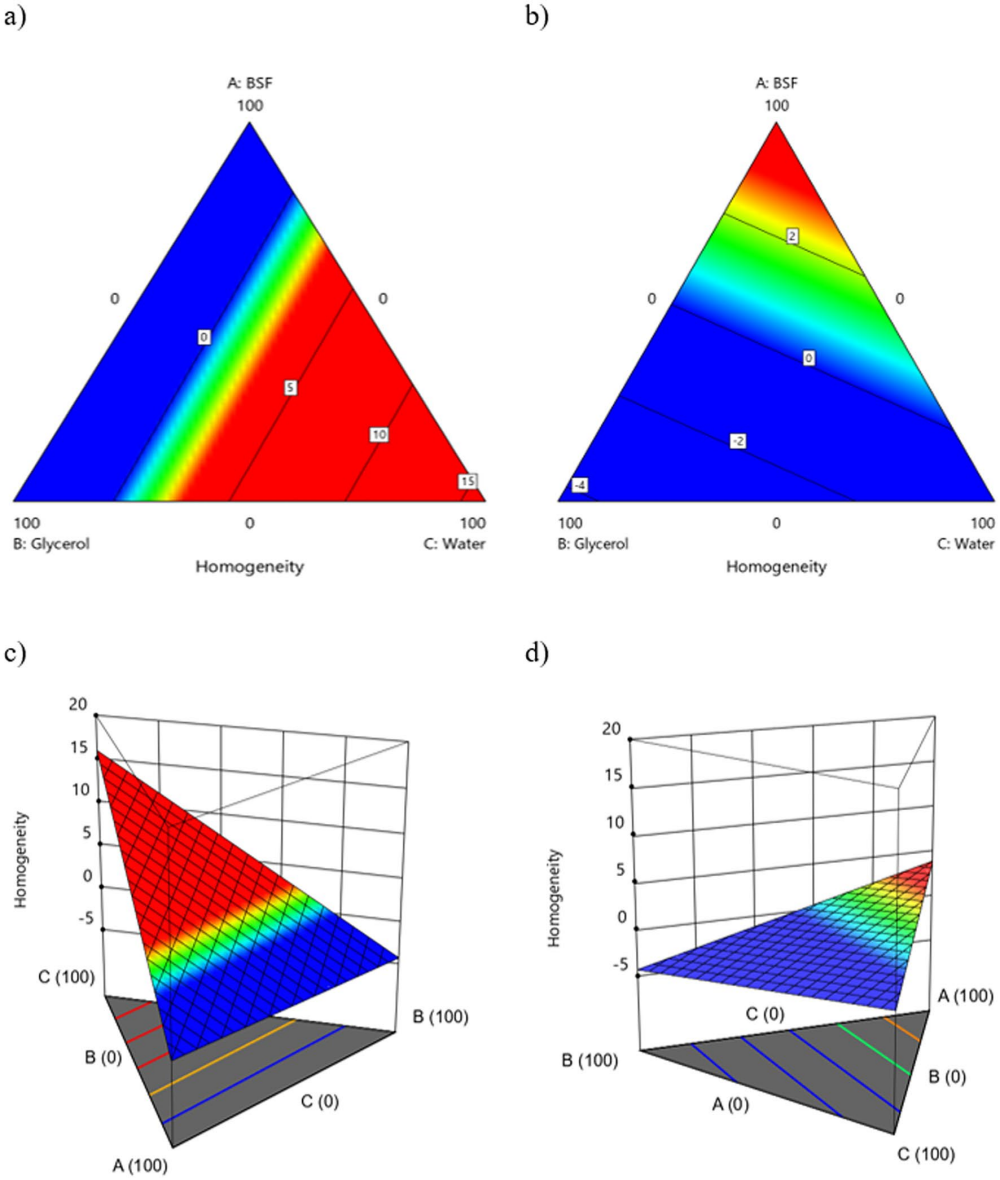
Contour plots for the response Homogeneity (**a**) S1-2D; (**b**) S2-2D; (**c**) S1-3D; (**d**) S2 – 3D.

**Figure 8:**
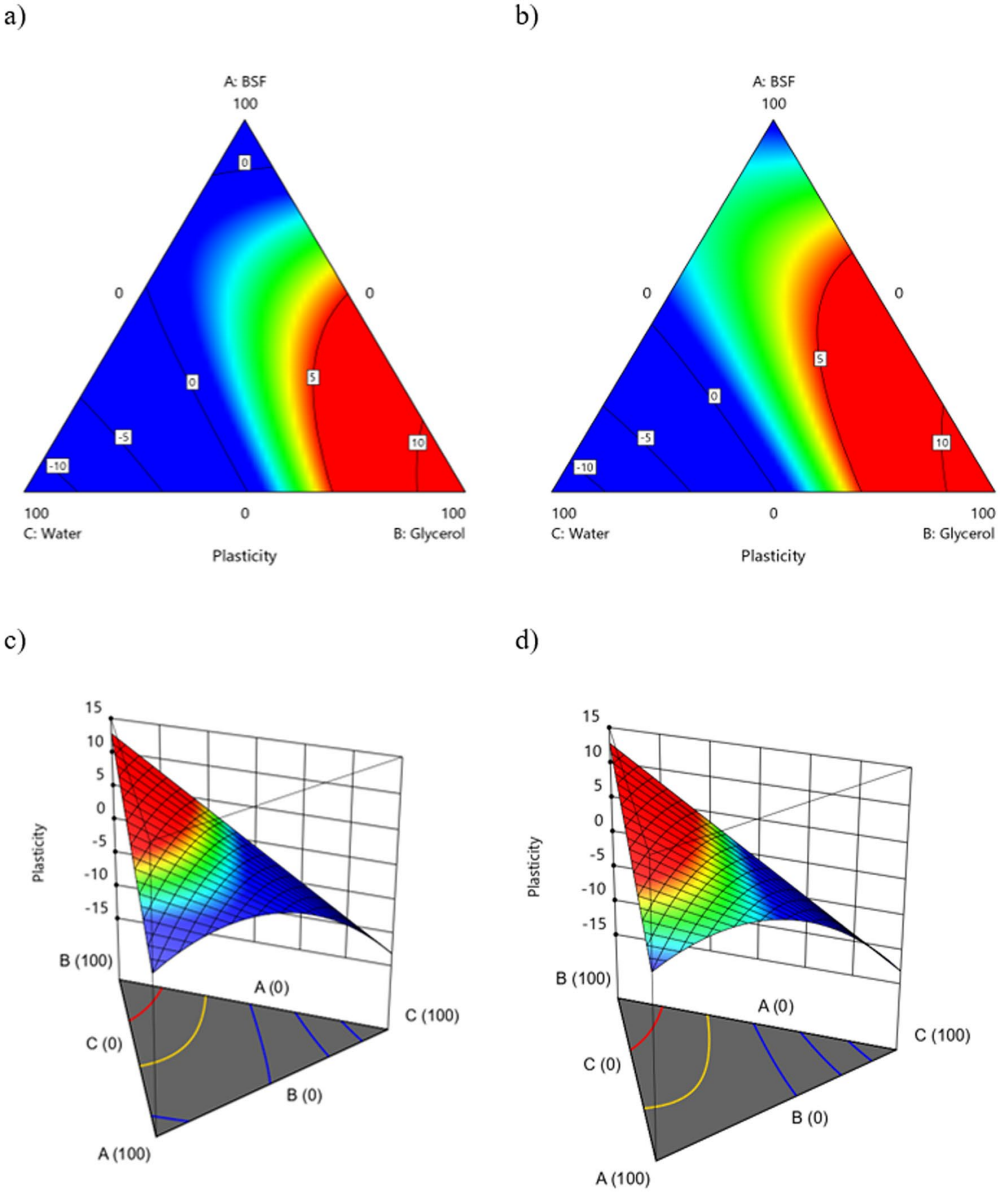
Contour plots for the response Plasticity (**a**) S1-2D; (**b**) S2-2D; (**c**) S1-3D; (**d**) S2-3D.

Models correlating factors (in single or in interaction) to panel test data are significant as confirmed by the p-value well below 0.05 and by the lack of fit well above 0.05, meaning the probability of data variation due to unknown factors is statistically irrelevant (Table 6). Furthermore, it is worth noting that only the mixture factors (BSF, glycerol and water) and the method of obtaining the nitrogen-rich fraction, S1 or S2, are significant factors for the evaluated responses. Subsequently, the particle size of the powder is not significant and for this reason it was not considered in the rest of this work. Furthermore, it is important to note that quadratic (interaction) terms cannot be neglected for the modeling of the responses. R^2^, Adjusted R^2^ and Pred-R^2^ (Table 6) confirm the good fit of the data and a fairly discrete predictive power of the models, with an important exception relating to the *Plasticity* response, which instead shows very high parameters (R^2^ = 0.93, Adjusted-R^2^ = 0.90 and Pred-R^2^ = 0.81). The fair predictive power related to *Homogeneity* and *Adhesion* is coherent with (i) the use of biological material (ii) the use of panel test (iii) the use of a lower number of levels for the panel test compared to *Plasticity*^53^. The estimation of the influence of independent factors (in single or in interaction) on the responses has been described in Table 7, Figs. 6, 7 and 8 where the coefficients of the coded equation of each model are reported as well as the contour plots for better understanding of the interactions between different factors. In contour plots, the red area indicates the combination of factors that would increase the selected response, while the blue area concerns the factors that alone or in interaction would strongly decrease the response.

For *Adhesion* the greatest influence is to be attributed to the interaction between glycerol and the method used to obtain the N-rich fraction, in particular their combined effect has a negative impact on the response, therefore it is recommended to use a moderate amount of glycerol together with S2 powder (+ 1 level of the factor N-rich obtainment), with the aim of improving adhesion (Fig. 6b,d). The best conditions to improve this response are given by using S1 with a blend that should include a limited amount of BSF (less than 60%), as shown in Fig. 6a,c.

For the response *Homogeneity*, once again an interaction factor must be carefully considered, as it is the one that has the greatest influence on this response: Water—N-rich fraction obtainment (Table 6). In strong similarity to the *Adhesion* response, a negative effect is still expected when a synergic interaction between these two factors is achieved. A limited amount of water must be used together with the S2 powder (+ 1 level of the factor N-rich obtainment), with the aim of improving the *Homogeneity* of the coating (Fig. 7b,d). On the contrary, with S1 at least 40% of Water must be used to reach the best results in terms of *Homogeneity,* as it promotes a wider response surface suitable for our purposes, the maximization of *Homogeneity* (red area). This result can be explained by considering the morphology of the different powders; As detailed in Fig. 4, the S1 powder shows a strongly irregular shape and size of the particle, leading to the need to use more water (which is the solvent in these coating formulations) to achieve a homogenous appearance of the coating. On the other side, S2 powder has a regular round morphology for which a limited amount of water is required to optimize the *Homogeneity* of the coating.

Regarding *Plasticity*, a strongly positive interaction effect has been calculated between the quantity of water and BSF, however their single effect on this property is negative for both (Table 6). In other words, only a very tailored proportion of BSF and water in the coating formulation can improve this property. This result suggests that water not only acts as a solvent but also supports the polymerization process, thus possibly limiting the needs for plasticizer in the mixture to achieve good plasticity of the coating. In fact, according to previous literature, the addition of water in combination with other plasticizer, such as glycerol, increases polymer-water interactions at the expense of polymer–polymer interactions, affecting the hydrogen bond or hydrophobic interactions^67,68^. As expected, the amount of glycerol, as a single factor, plays an important role on *Plasticity*, having a coefficient equal to 12.83, as this compound was included as a plasticizer in the coating formulation. These trends can be clearly seen in Fig. 8, where a very similar response surface can be observed by comparing the two methods of obtaining the nitrogen-rich fractions S1 and S2. In fact, as shown in Table 7 the obtainment of the N-rich factor plays a limited role for this property, having a coefficient of 1.26 only in interaction with the amount of BSF.

**Figure 9:**
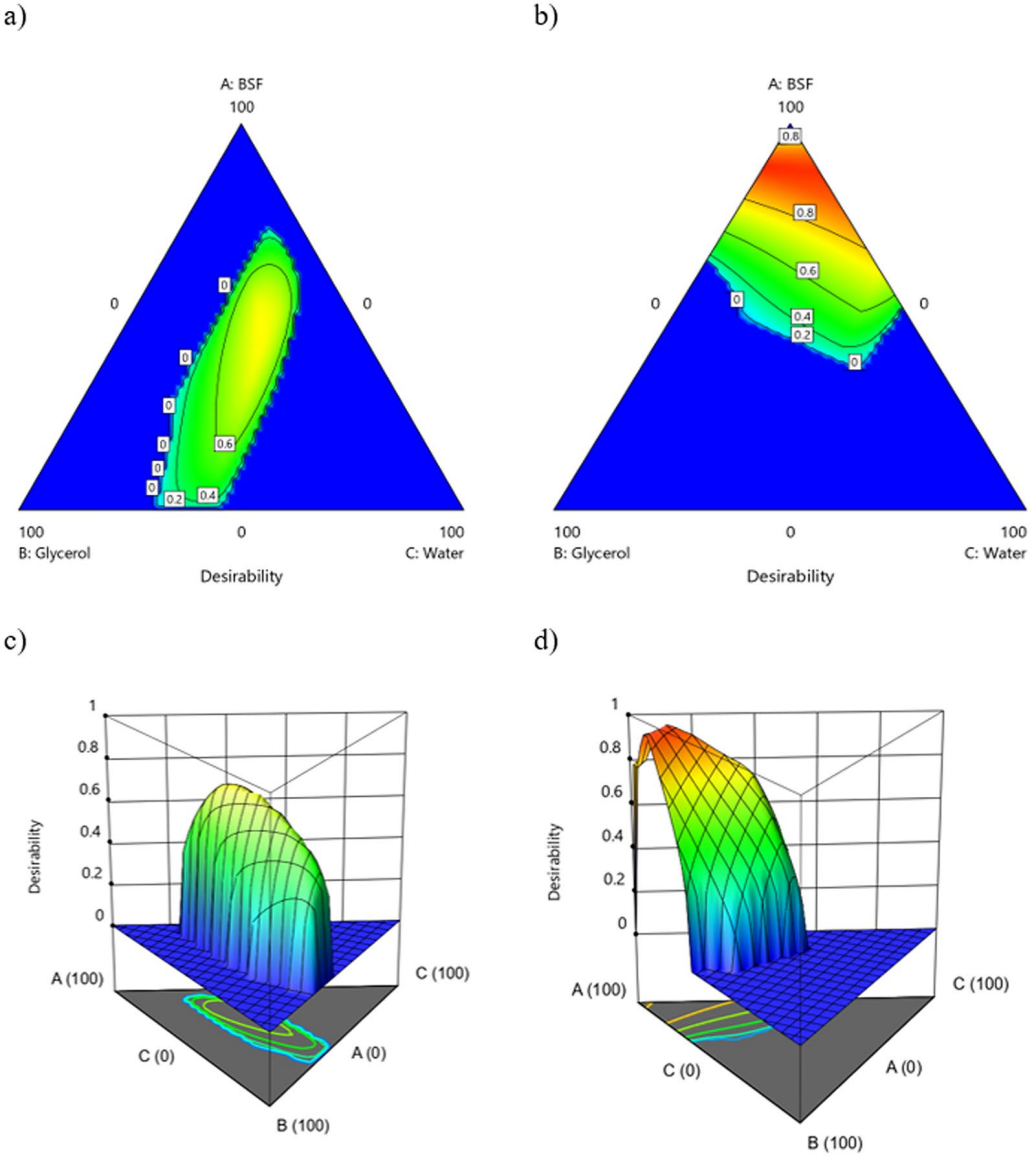
Contour plot for the Desirability function (**a**) S1-2D; (**b**) S2-2D; (**c**) S1-3D; (**d**) S2-3D.

From the results of the ANOVA analysis, it appears clear that further analysis is required in order to find a unique coating formulation, capable of optimizing the coating production, taking into account all the studied responses simultaneously. According to the method described in Sect. 2.3 a desirability function was set using the targets shown in Table 3. The graphical results of the desirability function were shown in the contour plots represented in Fig. 9. From this result, it can be concluded that the maximum quality of the final coating can be obtained using the S2 method for the N-rich fraction, considering the only red area of Fig. 9b,d. Also, it can be seen that a high amount of BSFL is required, therefore, for this coating the highest possible nitrogen release could be expected. In addition, it does not seem that a major difference would arise by using glycerol or water for the rest of the formulation, and this is a very positive result in terms of eco-friendly coating, as water can totally replace glycerol. Finally, the numerical optimization suggests that a formulation containing 89 wt.% of BSFL, obtained with the S2 method, and 11 wt.% of water could be the best compromise from an eco-compatible perspective, with the aim to avoid any synthetic compounds such as glycerol. This relevant result seems to overcome the limitations presented in previous literature, according to a plasticizer must be provided to consolidate BSF powder^49^. In addition, a formulation including near 90% of BSF could be a promising pathway to improve the overall amount of organic waste to be bio-digested, thereafter, promoting a strongly ecological way to produce fertilizers, in addition to other consolidated employment of N-rich BSF fraction such as fishmeal^69,70^.

### Characterization of the best and worst coatings

**Figure 10:**
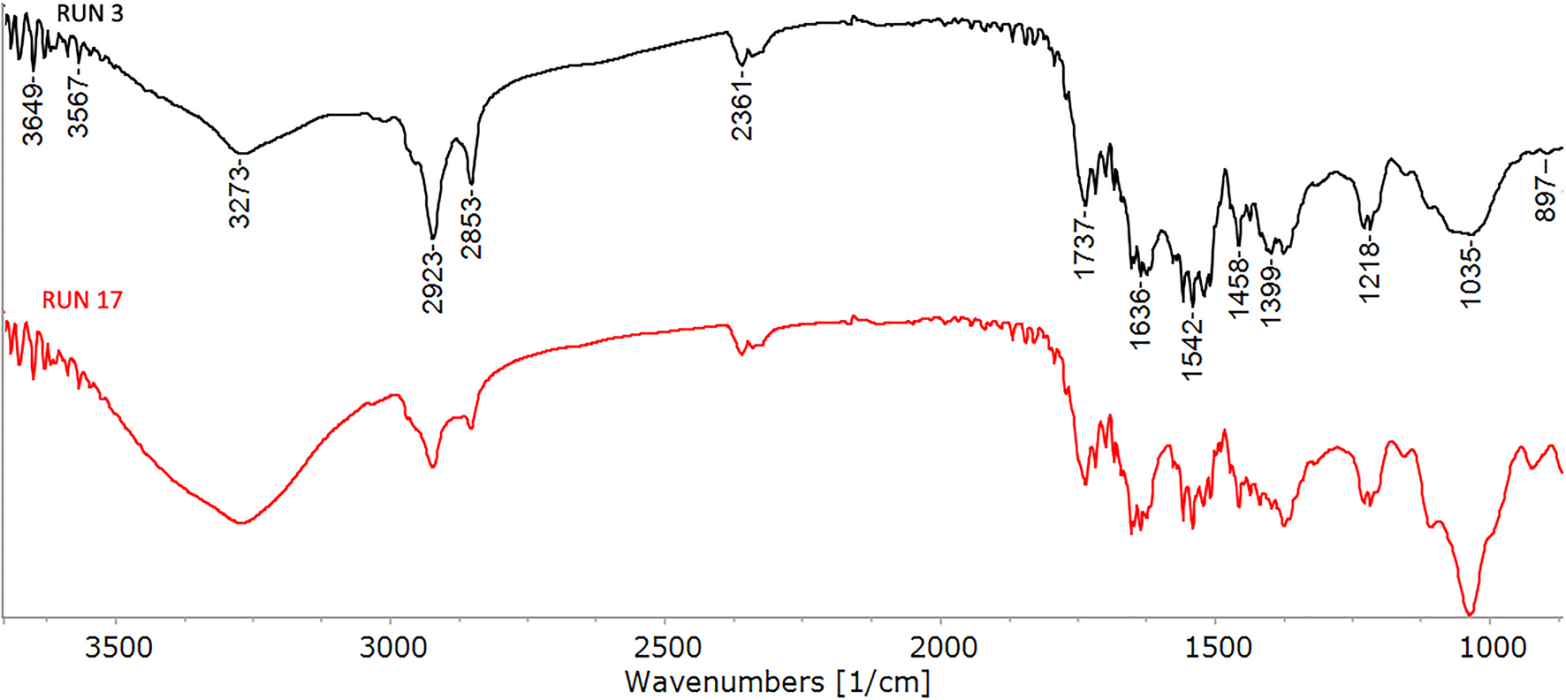
Normalized FT-IR transmittance spectra of the coatings related to RUN 3 and RUN 17.

Taking into consideration the results of the statistical analysis, a further characterization and comparison was carried out on two RUNs (Table 2) representative of the best and worst coating. In particular, RUN 17 was selected as representative of the best coating and RUN 3 as the worst. As shown in Fig. 10 the normalized FT-IR transmittance spectra of the two coatings were analyzed and similar spectra were obtained but with slight differences related with their formulation. At higher wavelengths it is possible to detect peaks related to the glycerol content in both spectra, in particular, at about 3650—3550 cm^−1^ O–H stretch group related to a hydroxyl/alcohol molecule type can be detected^71^. This result is consistent with the fact that the amount of glycerol is the same in RUN 3 and RUN 17 formulations. The other peaks can be identified with BSFL as detailed in paragraph 3.1, however, some differences emerge between the two spectra, due to the different N-rich fraction used and its interaction with water. As shown in Fig. 10, the peaks related to lipid residues (2923 cm^−1^ and 2853 cm^−1^) are more pronounced in the RUN 3 spectrum, as the presence of water in this coating formulation probably leads to partial immiscibility of the lipid residues, which are subsequently more evident in the FT-IR spectrum of the coating, compared to the powder. The peaks related to the chitin fraction such as the one at 3273 cm^−1^ (N–H stretch) and the one at 1035 cm^−1^ (C⎼O⎼C asymmetric stretch) are instead more pronounced in RUN 17, with respect to RUN 3, since it contains more N-rich fraction of BSFL in its coating formulation.

**Figure 11:**
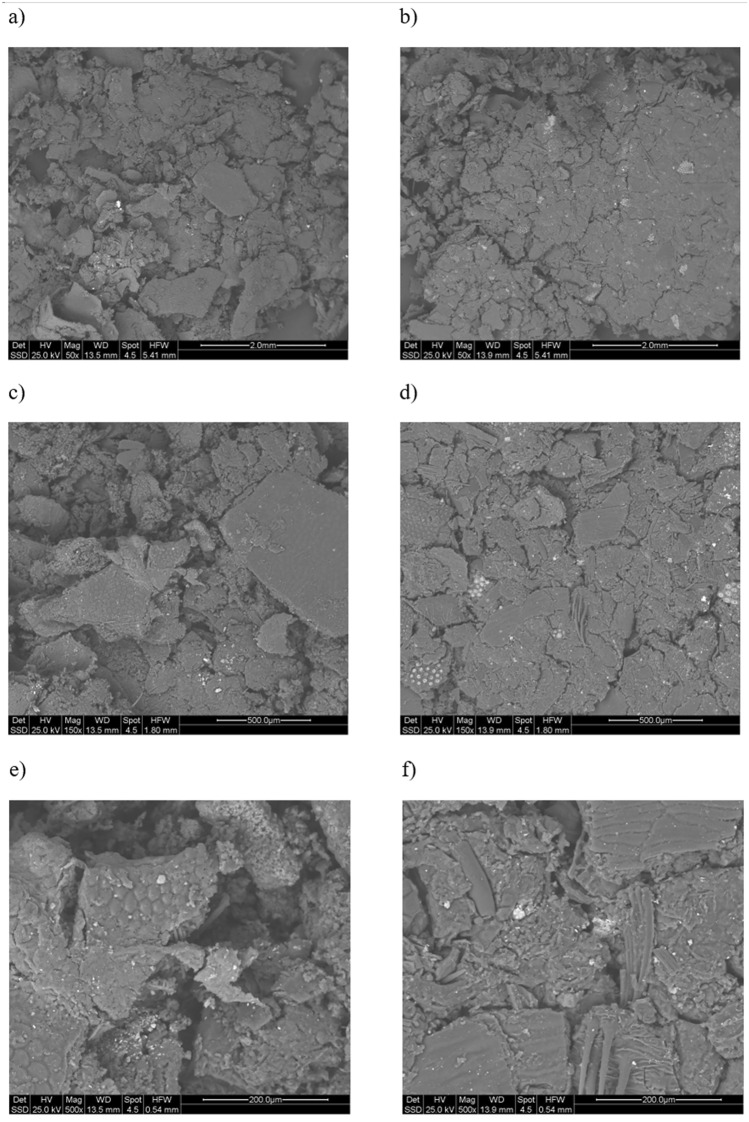
ESEM micrographs of the coatings related to RUN 17 and RUN 3 at different magnifications: (**a**) RUN 3—50X; (**b**) RUN 17- 50X; (**c**) RUN 3—150X; (**d**) RUN 17—150X; (**e**) RUN 3—500X; (**f**) RUN 17—500X.

Figure 11 shows the ESEM micrographs of the coatings obtained with the formulations indicated as RUN 17 and RUN 3. As can be seen, even from the different magnification used, the morphology of the RUN 3 coating is more heterogeneous, and, therefore, the coating is less compact than RUN 17, confirming the result obtained from the statistical analysis. In particular, there are some cracks in the coating relating to RUN 17 (Fig. 11b,d), while strong and clear separations can be observed between the different parts of the coating for RUN 3 (Fig. 11a,c). At the highest magnification (Fig. 11e,f) it is possible to observe that, even in the case of a cracked part of the coating, in RUN 17 (Fig. 11f) the separated parts of the coating are quite homogeneous in shape, morphology and size, while for RUN 3 (Fig. 11e) very heterogeneous parts of the coating can be observed.

## Conclusions

The present paper investigated the possibility of using organic biomass derived from the bioconversion through insects (BSFL) of vegetable by-products from industrial activities in the formulation of coatings for core–shell NPK fertilizers. First, it has been assessed that vegetable by-products are more favorable to the rearing of insects than manure, according to the current European and Italian legislation. As a further result of this investigation, the use of vegetable by-products for BSFL rearing is able to guarantee at least the same nitrogen content (~ 7%) in organic biomass compared to manure, opening up new concrete possibility for the virtuous disposal of vegetable by-products. Finally, through statistical methods, it was possible to calculate the best coating formulation based almost entirely on BSFL nitrogen-rich fraction (89%) and avoiding the presence of synthetic plasticizer such as glycerol, with a view to circular economy. As future perspective, the employment of this fertilizer could be studied in terms of Life Cycle Assessment and Life Cycle Cost analysis to evaluate numerically its beneficial effect in a circular economy perspective. In addition, a tailored bioconversion strategy could be implemented in order to promote the nitrogen-enriched fraction amount by considering pollutants in organic wastes such as industrial flocculants and particular conditions such as controlled moisture or artificial light.

## Data Availability

The datasets generated and analyzed during the current study are not publicly available due to funding policy but are available from the corresponding author on reasonable request.
